# Validation of the Iranian version of the childbirth experience questionnaire 2.0

**DOI:** 10.1186/s12884-019-2606-y

**Published:** 2019-12-04

**Authors:** Solmaz Ghanbari-Homayi, Anna Dencker, Zahra Fardiazar, Mohammad Asghari Jafarabadi, Sakineh Mohammad-Alizadeh-Charandabi, Shahla Meedya, Eesa Mohammadi, Mojgan Mirghafourvand

**Affiliations:** 1 Department of Midwifery, Faculty of Nursing and Midwifery, Tabriz University of Medical Sciences, Tabriz, Iran; 2 Institute of Health and Care Sciences, Sahlgrenska Academy, University of Gothenburg, Gothenburg, Sweden; 3 Women Reproductive Health Research Center, Tabriz University of Medical Sciences, Tabriz, Iran; 4 Department of Statistics and Epidemiology, Tabriz University of Medical Sciences, Tabriz, Iran; 5 Road Traffic lnjury Research Center, Tabriz University of Medical Sciences, Tabriz, Iran; 6 Social determinants of Health Research Center, Tabriz University of Medical Sciences, Tabriz, Iran; 7 PhD, Senior Lecturer, Member of South Asia Infant Feeding Research Network (SAIFRN), School of Nursing, Faculty of Science, Medicine and Health, University of Wollongong, Wollongong, Australia; 8 Department of Nursing, School of Medicine, Tarbiat Modares University, Tehran, Iran

**Keywords:** Birth experience, Birth satisfaction, Childbirth experience questionnaire, Validity, Reliability, Psychometric, Iran

## Abstract

**Background:**

Assessing women’s childbirth experiences is a crucial indicator in maternity services because negative childbirth experiences are associated with maternal mortalities and morbidities. Due to the high caesarean birth rate in Iran, measuring childbirth experience is a top priority, however, there is no standard tool to measure this key indicator in Iran. The aim of present study is to adapt the “Childbirth Experience Questionnaire 2.0” to the Iranian context and determine its psychometric characteristics.

**Methods:**

Childbirth Experience Questionnaire 2.0 was translated into Farsi. A total of 500 primiparous women, at 4 to 16 weeks postpartum, were randomly selected from 54 healthcare centres in Tabriz. Internal consistency and reliability was calculated using the Cronbach’s Coefficient alpha and Intraclass Correlation Coefficient, respectively. Construct validity was assessed using exploratory and confirmatory factor analysis and discriminant validity using the known-group method and the Mann-Whitney U-test.

**Results:**

The internal consistency and reliability for the total tool were high (Cronbach’s alpha = 0.93; Intraclass Correlation Coefficient = 0.97). Explanatory factor analysis demonstrated the adequacy of the sampling (Kaiser-Meyer-Olkin = 0.923) and significant factorable sphericity (*p* < 0.001). Confirmation factor analysis demonstrated acceptable values of fitness (RMSEA = 0.07, SRMSEA = 0.06, TLI = 0.97, CFI > 0.91, *x* ^2^/ df = 4.23). Discriminatory validity of the tool was confirmed where the CEQ score and its subdomains were significantly higher in women who reported having control over their childbirth than women who did not.

**Conclusion:**

The Farsi version of the Childbirth Experience Questionnaire 2.0 tool is a valid and reliable tool and can be used to measure the childbirth experience in Iranian women.

## Background

Maternal and neonatal mortality and morbidity rates are generally used as a standard indicator to evaluate midwifery care services. Recently, maternal satisfaction with the midwifery and childbirth services has been introduced as a healthcare quality index. Since maternal satisfaction has an interdependent relationship with childbirth experiences, exploring women’s needs and expectations are important to enhance the quality of care [1–3]. Women remember their childbirth experience for years, which may have many short-term and long-term effects on women’s health [4]. Negative childbirth experiences can increase the incidence of postpartum depression, fear of childbirth, unwillingness of future childbearing, choosing Caesarean section over a vaginal delivery, and poor breastfeeding outcomes [5–8]. To enable healthcare providers and policy-makers in providing supportive services according to maternal needs [9, 10], there is a need for reliable and valid tools.

Different instruments have been developed to measure the childbirth experience, however many of them have looked at only one dimension of the childbirth experience. For example, The Labour Agentry Scale (LAS) only covers the control concept of the birth experience [11]. The Wijma Delivery Expectancy/Experience Questionnaire (W-DEQ) measures the fear of childbirth [12]. The Labour and Delivery Satisfaction Index focuses on the specific psychometric properties [13] and The Maternal Satisfaction Scale covers only one dimension of childbirth experience during caesarean section [14]. The Childbirth Perception Scale includes 12 items, of which six items evaluate the childbirth experience immediately after birth and the remainder evaluate maternal perception of childbearing at first week postpartum. Since this tool evaluates women’s experience of birth immediately following childbirth at which stage, the positive experience of having a healthy child is at its peak, the likelihood of reporting a ‘falsely’ positive childbirth experience is a limitation of this tool [15, 16]. The Childbirth Trauma Index (CTI) was developed to address adolescents [17]. The Pregnancy and Maternity Care Patients’ Experiences Questionnaire assesses maternal experience of care services provided during pregnancy, childbirth, and postpartum by healthcare centres. The Responsiveness in Perinatal and Obstetric Health Care Questionnaire measures the quality of prenatal care. Since the childbirth experience is a multidimensional concept and is not limited to services provided by healthcare providers, this can be a limitation for these tools [18–20].

The experience of labour and childbirth are multidimensional concepts, therefore, the Childbirth Experiences Questionnaire version 2.0 (CEQ 2.0) measures the multidimensional childbirth experience of primiparous women. The CEQ was developed by Dencker et al. (2010) and included 22 items with four domains (‘Professional Support’, ‘Participation’, ‘Own Capacity’ and ‘Perceived Safety’). Nineteen items are scored based on the 4-point Likert Scale and 3 items are scored between 0 to 100 using a visual analogue scale (VAS). Results of psychometric properties of the original study showed that CEQ is a valid and reliable tool. The CEQ has been validated in the UK population [21] and used in several research papers [22–24]. The revised edition of this tool (CEQ 2.0) included 23 items. Some items from the CEQ have been removed (including questions from the ‘Professional Support’ and ‘Participation’ domains) and new items have been added. Some items have been reworded and some items are entirely new. CEQ 2.0 covers four areas, namely ‘Own Capacity’ (items 1, 2, 4, 5, 6, 7, 21, and 22), ‘Professional Support’ (items 11, 13, 14, 15, and 16), ‘Perceived Safety’ (items 3, 17, 18, 19, 20, and 23), and ‘Participation’ (items 8, 9, 10, and 12). Twenty items are scored based on the 4-point Likert Scale (“totally agree”, “mostly agree”, “mostly disagree”, “totally disagree”) and 3 items are scored between 0 to 100 [(0–40 = 1); (41–60 = 2); (61–80 = 3); (81–100 = 4)] using a visual analogue scale (VAS) (Additional file 1). The items of negative experience (experience of pain, sense of tiredness, sense of fear, negative memories, and memories causing depressive thoughts) are negatively scored. Item ratings are aggregated to scale scores by summing the coded values of the items in each scale and dividing by the number of items in that scale; higher scores indicate a more positive childbirth experience [20, 21]. Based on the Terwee’s criteria (a quality criteria’s checklist for measurement properties of health field scales), the CEQ-2 psychometric properties quality score has been shown to be a suitable tool for clinical studies [25] and it has been translated into many languages such as Spanish [26], Malaysian [27] and Danish [28].

In Iran, due to increased rate of caesarean section which is associated with maternal fear and other aspects of birth experience [29], there is a need to measure women’s experience with a standard psychometric tool. This study is aimed at investigating the psychometric properties of the CEQ-2 that can be suitable for Iranian women.

## Methods

We used a few stages to develop and test the Farsi version of the tool. The first stage was to translate the tool and pilot for face validity, content validity and reliability of the tool. The next stage involved the evaluation of the psychometric properties with a large sample size for construct validity.

### Translation procedure

Fourteen items common to CEQ from earlier translation work by Professor Abbaspoor and colleagues (Ahvaz University of Medical Sciences, Iran) were used in CEQ 2.0. The remaining 9 items in CEQ 2.0 were translated from English into Farsi by two female professional translators, native in Farsi and very skilled in English, in two separate translations. These translations were reviewed by the research team, compared with each other, contradictions were corrected, and a Farsi version was created by integrating both translations. Then, the Farsi version was back-translated into English by two translators, native in Farsi and very skilled in English. The back-translators were not familiar with the CEQ questionnaire. The back-translation was very close to the original English CEQ. The translated Farsi version was reviewed by two experts (one expert in translation of questionnaire and one familiar with the concepts) (Additional file 2). The Farsi version was evaluated by four women about simplicity and clearness. All four women found the items of CEQ 2.0 simple and easy to understand.

### Face validity

Face validity was assessed qualitatively based on the opinions of 10 experts in the fields of Midwifery, Reproductive Health, Obstetrics and Gynecology, Clinical Psychology, Nursing and Tool Development, who were asked to comment on the simplicity, transparency and relevance of the translated items. The items were then corrected in terms of use of appropriate and transparent vocabulary, grammar, and importance of items based on their context in Iran. In a pilot test, 20 women answered the CEQ 2.0 in the postpartum period and were asked to comment on its simplicity in terms of understanding, relevancy, and ambiguity of the items. According to their opinions, no further changes were necessary. Face validity was also quantitatively measured using the item impact method based on the women’s opinions. To this end, the items were scored based on a 4-item Likert scale anchored by 4 (very important) to 1 (not important at all). Then, the impact score was obtained using the following formula (Impact Score = Frequency (%) × Importance). Frequency reflects the number of respondents who scored the items a 4, and importance reflects the mean score. An impact score higher than 1.5 was considered valid [30].

### Content validity

The content validity was obtained based on expert opinions, Content Validity Ratio (CVR) and Content Validity Index (CVI) values. A checklist with two parts was designed for each expert. The first and second parts of the checklist were designed for calculation of CVI and CVR, respectively. The first part of the checklist assessed clarity, simplicity, and relevance of items based on a 4-point Likert scale. The second part assessed the necessity of each item based on a 4-point Likert scale from not useful to necessary. A CVR higher than 0.62 and CVI higher than 0.79 were considered valid [31].

### Reliability

Reliability was determined using the internal consistency test and test-retest reliability. The internal consistency was calculated using the Cronbach’s Coefficient alpha. A Cronbach’s alpha higher than 0.7 was considered reliable [31]. The test-retest reliability was calculated through test-retest of 20 eligible women with a two-week interval and the calculation of Intra Correlation Coefficient (ICC). An ICC between 0.6 and 0.8, and higher was regarded as good and excellent, respectively [30].

### Study participants

This study enrolled primiparous women, aged at least 18-years-old, with cephalic presentation at the gestational age of 38–42 weeks undergoing a vaginal childbirth. Women with obstetric problems, such as placenta previa or placental abruption, elective or unplanned caesarean section, mental disability, deaf-mute, history of depression during pregnancy or postpartum depression, maternal report of using antidepressants, and major congenital anomalies, were excluded.

### Ethical consideration

The study protocol was confirmed by the Ethics Committee of Tabriz University of Medical Sciences (code: IR.TBZMED.REC.1396.786). All participants signed the informed written consent form. For illiterate participants, their fingerprints were taken after oral presentation of information.

### Recruitment and data collection

First, 44 urban health centres and 10 rural health centres were selected among the total urban (87 centres) and suburban (15 centres) health centres in Tabriz. Then, women who had a vaginal childbirth at least 4 weeks and maximum 16 weeks prior were identified as eligible from each health centre. Next, a list of mothers in each health centre was prepared based on their electronic medical records. The required sample size for each centre was determined using the proportional to size method and the participants were randomly selected. The researcher contacted the selected mothers and invited them to participate after explaining the research objectives and confidentiality of their information. In a 15–20-min meeting with each participant, the socio-demographic and CEQ questionnaires were completed by the researcher. The obstetrics information was extracted from the participants’ medical records after obtaining their permission.

### Sample size

For purification of the assessment tool in factor analysis, Nunnally & Bernstien (1994) recommended a minimum sample size of 10 per item [32]. As a result, the initial sample size was estimated to be 250; however, due to the use of cluster sampling and application of design effect of 2, the sample size was increased to 500.

### Statistical analyses

Data were analysed using SPSS Statistics for Windows version 25.0 (IBM Inc., Armonk, NY, USA) and STATA software [ver.15] (StataCorp, College Station, Texas 77,845 USA). Construct validity was assessed by a) exploratory factor analysis; b) confirmatory factor analysis; and c) discriminant validity which was evaluated by the known-groups method.

### Exploratory factor analysis

Scale-based EFA was performed for each scale separately. The exploratory factor analysis was assessed by the Kaiser-Meyer-Olkin (KMO) and Bartlett’s test of sphericity for each separate scale. Values higher than 0.7, along with significance of test confirms the adequacy of the exploratory factor analysis [33]. Moreover, the Eigen value and Scree Plot were used to determine how many factors should be retained for the tool. The second stage of the scale-based exploratory factor analysis, including factor rotation, was mathematically calculated. The goal of this stage was to make the factor constructs simple and interpretable. One way to achieve a simple structure in the scale-based exploratory factor analysis is using the Principal Axis Factoring (PAF) for extracting factor and oblimin rotation (with delta value of zero and Kaiser normalization). The correlated items were summarized into new variables, called factor. After the extraction of factors, each of them was named based on the variables (items) of each factor. If the Principal Axis Factoring of a factor is lower than 0.3, it is poorly correlated with the extracted set of factors and may be removed [34].

### Confirmatory factor analysis

To assess the structure of factors obtained from the exploratory factor analysis, the model was fitted using the confirmatory factor analysis. The factor analysis investigates the confirmation of the exploratory model theoretically and the relationship between factors. The fitness of indices was used to evaluate the model fitness. To confirm the model by these indices, Root Mean Square Error of Approximation (RMSEA) was considered lower than 0.08, Standardized Root Mean Square Error of Approximation (SRMSEA) < 0.08, Comparative Fit Index (CFI) ≥ 0.90, Tucker- Lewis Index (TLI) ≥ 0.95, Normed chi-square (*x* ^2^/ df) < 5.0 [34, 35].

### Discriminant validity

The discriminant validity was assessed using the known-group method and the independent Mann-Whitney U-test to investigate the intergroup difference in overall scores of childbirth experience and its subdomains by labour duration [20, 36], oxytocin augmentation [37], and the sense of control over childbirth [38]. Sense of control over birth was measured by a question “Did you feel you had control on your labour and childbirth?” with the response options of Yes (1) or No (0). According to some studies into the childbirth experience, it is expected that women with shorter labour, without oxytocin augmentation, and those who reported sense of control over childbirth have a better childbirth experience. The effect size was determined based on the Cohen’s definition (the mean difference between the two groups, and then dividing the result by the pooled standard deviation) [39]. The values between 0.2 and 0.5, between 0.5 and 0.8, and higher than 0.8 were considered low, moderate, and high, respectively [40].

## Results

A total of 697 eligible women were identified through records in health centers and from them, 500 primiparous women (72%), during postpartum period (passing of at least 4 weeks and maximum 16 weeks of their childbirth), agreed to participate in the study and were enrolled between May and August 2018.

### Participants’ characteristics

The mean age of the participants was 23.5 years. A quarter of the participants experienced labour which lasted over 12 h. Almost all participants underwent episiotomy. Participants’ characteristics are presented in Table 1.

**Table 1:** Characteristics of the study participants (*n* = 500)

Variables	Number (%)
Maternal age^a^ (years)	23.5 (4.8)
Education	
High school or below	412 (82.4)
College or above	88 (17.6)
Occupation	
Home maker	458 (91.6)
Employed	34 (6.8)
Student	8 (1.6)
Gestational age^a^ (week)	39.0 (1.3)
Abortion history	84 (16.8)
Labour duration more than 12 h	127 (25.5)
Oxytocin augmentation	331 (66.2)
Episiotomy	494 (98.8)
Sense of control during childbirth	266 (53.2)
Hospital Type	
Public	360 (72)
Private	80 (16.0)
Organizational	60 (12)
Completion time of the CEQ (weeks)^a^	10.1 (4.4)

^a^Mean (SD)

### Face validity

All items in the tool were reported easily understandable and transparent (*n* = 20 primiparous women). The impact score of each item varied between 3.0 and 4.0 (*n* = 10 expert) (Table 2).

**Table 2:** The impact Score, CVI, and CVR for CEQ 2.0 (*n* = 10 experts)

Items	Impact Score	CVI	CVR
	*n* = 20 mothers	*n* = 10 experts	
CEQ1	3.7	0.90	0.80
CEQ2	3.3	0.83	0.80
CEQ3	4	1	1
CEQ4	3.7	1	1
CEQ5	3.3	0.96	1
CEQ6	4	0.96	0.80
CEQ7	3.5	0.90	1
CEQ8	4	1	1
CEQ9	4	1	1
CEQ10	3.5	0.90	0.80
CEQ11	3.7	0.93	1
CEQ12	4	0.96	1
CEQ13	4	1	1
CEQ14	3.7	1	1
CEQ15	3.7	0.96	0.80
CEQ16	3.0	0.90	0.80
CEQ17	3.5	0.96	0.80
CEQ18	4	1	1
CEQ19	4	1	0.80
CEQ20	4	1	1
CEQ21	4	1	1
CEQ22	3.7	0.90	0.80
CEQ23	3.7	1	0.80

### Content validity

The calculated CVI and CVR values were in the range 0.83–1.00 and 0.80–1.00, respectively (*n* = 10 experts) (Table 2).

### Reliability

The overall Cronbach’s alpha was 0.93 (*n* = 20 primiparous women). Cronbach’s alpha of Own Capacity, Participation, Professional support, and Perceived safety was 0.87, 0.67, 0.88, and 0.86, respectively. The overall intraclass correlation coefficient (ICC) of CEQ-2 was higher than 0.9, indicating that the test-retest reliability was acceptable (Table 3).

**Table 3:** Cronbach’s alpha, Intraclass Correlation Coefficients and scale-based Factor loadings of the CEQ 2.0 (*n* = 500)

	Factor 1	Factor 2	Factor 3	Factor 4
Own capacity				
Labour and birth went as I had expected.	.781			
I felt strong during labour and birth.	.765			
I felt capable during labour and birth	.735			
I was tired during labour and birth.	.529			
I felt happy during labour and birth.	.803			
I felt that I handled the situation well.	.807			
As a whole, how painful did you feel childbirth was?	.499			
As a whole, how much control did you feel you had during childbirth?	.610			
Perceived safety				
I felt scared during labour and birth.		.461		
My impression of the team’s medical skills made me feel secure.		.624		
I have many positive memories from childbirth.		.911		
I have many negative memories from childbirth.		.936		
Some of my memories from childbirth make me feel depressed.		.768		
As a whole, how secure did you feel during childbirth?		.593		
Participation				
I wish the staff had listened to me more during labour and birth.			.489	
I could get up and move around as much as I wanted.			.478	
I took part in decisions regarding my care and treatment as much as I wanted.			.586	
I received the information I needed during labour and birth.			.836	
Professional support				
Both my partner and I were treated with warmth and respect.				.735
I would have preferred the midwife to be more present during labour and birth.				.738
I would have preferred more encouragement from the midwife.				.748
The midwife conveyed an atmosphere of calm.				.729
The midwife helped me to find my inner strength.				.810
% Variance Explained	42.8	7.2	9.8	4.6
	Total score = 64.6			
Cronbach’s alpha	0.87	0.86	0.67	0.88
	Total score = 0.93			
Intraclass Correlation Coefficient (95% CI)	0.98 (0.95 to 0.99)	0.99 (0.97 to 0.99)	0.81 (0.58 to 0.92)	0.97 (0.94 to 0.99)
	Total score = 0.97 (0.93 to 0.99)			
Mean (SD)	2.5 (0.8)	2.6 (0.8)	2.6 (0.8)	2.8 (0.9)

### Factor analysis

The factor analysis was calculated with 500 primiparous women. The KMO (0.923) and Bartlett test (*p* < 0.001) confirmed the adequacy of the scale-based exploratory factor analysis. Regarding the moderate and high correlation (> 0.3) between the extracted factors, the use of oblimin rotation was confirmed. All items had factor loadings of higher than 0.3 and thus were maintained in the questionnaire.

Since Factor 1 had the highest eigenvalue (9.86), it produced a better prediction (42.8%) of the childbirth experience than other factors. According to the explained variance index, the prediction power of the tool was 64.6%. In other words, 64% of the changes were predicted by four factors extracted based on the exploratory analysis. The matrix of extracted factor loadings from the scale-based factor analysis is presented in Table 3.

**Fig. 1:**
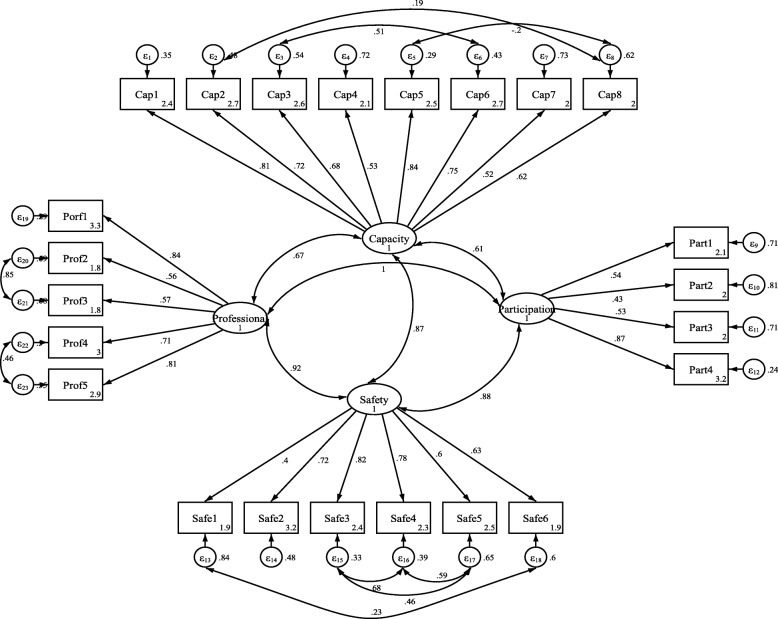
CFA factor loading

The fitness of the confirmatory factor analysis was required for evaluation of the factor structures. Acceptable values of fitness indices indicated good model fitness (RMSEA = 0.07, SRMSEA = 0.06, TLI = 0.97, CFI > 0.91, *x* ^2^/ df = 4.23) (Table 4). Figure 1 shows the flow diagram of the model of the four factors the Persian version based on the CEQ 2.0. The minimum and maximum coefficients of item-scale relationship were 0.43 and 0.87. Moreover, all coefficients of item-scale relationship in the confirmatory factor analysis were significant (*p* < 0.001), that all items were significantly correlated with their factor.

**Table 4:** Confirmatory factor analyses: fit Index CFA of CEQ 2.0 (*n* = 500)

Models	X^2^	df	$$ \raisebox{1ex}{${x}^2$}\!\left/ \!\raisebox{-1ex}{$ df$}\right. $$	RMSEA (90%CI)	SRMR	CFI	TLI
Model 1	910.9	250	4.23	0.07 (0.07 to 0.08)	0.06	0.91	0.97

*x*^*2*^*/df* Normed chi-square; *RMSEA* Root Mean Square Error of Approximation, *SRMR* Standardized Root Mean Square Residual, *CFI* Comparative Fit Index, *TLI* Tucker- Lewis Index

### Discriminant validity

Discriminant validity was employed for measurement of the construct validity using the known-groups method. In women with shorter stay in the labour room (< 12 h), the overall CEQ 2.0 score and the subdomain scores of perceived safety and own capacity were significantly higher than women with longer stay (> 12 h), with very small effect sizes. There was no significant difference in the overall CEQ 2.0 score and its subdomains between women with or without oxytocin augmentation during labour (*P* = 0.874). The CEQ 2.0 score and all subdomain scores were significantly higher in women who reported having control over their labour and childbirth than women who did not (*P* < 0.001), with large effect sizes (Table 5).

**Table 5:** CEQ 2.0 overall and sub scales scores by different groups (*n* = 500)

Variables	Own capacity	Participation	Professional support	Perceived safety	Total scale
	Mean (SD)				
Duration of labour≤12 h (*n* = 371)	2.6 (0.7)	2.7 (0.8)	2.8 (0.8)	2.7 (0.8)	2.7 (0.6)
Duration of labour> 12 h (*n* = 127)	2.3 (0.8)	2.6 (0.9)	2.6 (0.9)	2.4 (0.9)	2.4 (0.7)
*P*-value^*^	0.001	0.610	0.097	0.005	0.008
Cohen’s effect size	0.38	0.11	0.17	0.31	0.28
Oxytocin augmentation (*n* = 331)	2.5 (0.8)	2.6 (0.8)	2.8 (0.9)	2.6 (0.8)	2.6 (0.7)
No oxytocin augmentation (*n* = 169)	2.5 (0.7)	2.7 (0.7)	2.7 (0.8)	2.6 (0.8)	2.6 (0.6)
*P*-value^*^	0.852	0.429	0.081	0.998	0.912
Cohen’s effect size	0.06	0.13	0.09	0.00	0.01
Sense of having control over childbirth (*n* = 266)	3.0 (0.6)	2.9 (0.7)	3.1 (0.8)	3.0 (0.7)	3.0 (0.5)
Lack of sense of having control over childbirth (*n* = 234)	2.0 (0.6)	2.3 (0.8)	2.4 (0.9)	2.1 (0.7)	2.2 (0.6)
*P*-value^*^	< 0.001	< 0.001	< 0.001	< 0.001	< 0.001
Cohen’s effect size	1.73	0.79	0.76	1.23	1.44

^*^Mann-Whitney U-test

## Discussion

The results from this study provide evidence that the translated version of the CEQ 2.0 is a valid and reliable measure of childbirth experience among the representative sample of Iranian women in Tabriz. Results showed the clarity, simplicity, and relevance of the items and reliability of the tool at an acceptable level. The Farsi version of CEQ 2.0 had similar internal consistency with the original (Swedish) [20] and English versions [21], where the subscales professional support and participation had the highest and lowest internal consistency, respectively. In the Swedish version, the overall Cronbach’s alpha of the tool was not reported; however, the Cronbach’s alpha of the Farsi edition (0.93) was similar to the English edition (0.90). The reliability results of the Farsi and English editions were similar for the entire tool and its subdomains. Four factors with the prediction power of 64.6% were extracted based on the exploratory analysis. The CFA results showed acceptable fitness.

In the Swedish validation study [20], the subscale scores of CEQ were significantly higher in women with shorter labour and women without oxytocin augmentation of labour. In this study women with shorter time in the labour room also scored higher but with very small effect sizes. The mean subscales scores were lower than in Sweden, except for the own capacity subscales [20]. There are several differences between the childbirth context in Iran and Sweden and between the samples in both studies. For example, in the Swedish study all women had a spontaneous onset of labour. Instrumental birth accounted for approximately 12% in Sweden [20] and only 2 out of 500 participants in the present study and in the Swedish study also women with caesarean birth were included. In Iran, it is much more common that nulliparous women have and episiotomy (70%), than in Sweden (7%) [41, 42]. Dencker et al’s study was based on a prospective clinical study; whereas, the current study was a cross-sectional one. Therefore, contextual differences and differences between the samples may lead to differences in the results. A high proportion of women received oxytocin augmentation during labour in both studies, 67% [20].

In the Swedish study, the biggest effect sizes were seen when comparing spontaneous vaginal birth with instrumental birth (instrumental vaginal and caesarean); whereas, since women undergoing caesarean were not enrolled and only 2 out of 500 participants had instrumental vaginal delivery in the current study, this factor was not investigated.

In this study, there was no significant difference between women with shorter duration of labour in the subdomains of participation and professional support. This finding is consistent with the study conducted to evaluate psychometric components of the first CEQ among a Spanish population. Women with shorter labour attained higher scores in own capacity, perceived safety, participation subscales and an overall CEQ score than women with longer labour [26].

The CEQ score and its subdomains were significantly higher in women who reported having control over their labour and childbirth than women who did not. Furthermore, other studies have reported that having a sense of control over childbirth is an important predictor of birth experience [38, 43].

### Strengths and weaknesses

The larger sample size with homogeneity in ethnicity and marital status in addition to the random selection of the participants were the strengths of the current study. Another strength was using only one technique (interview) for data collection. Research data was selected between 1 and 4-month postpartum. The childbirth experience data collection time is important and can affect the way women report their childbirth experience. The underlying reason is that the administration of this tool during one-month postpartum may produce false positive or negative results as the mothers may still feel uncomfortable or joyful about having a healthy newborn [16, 20].

Regarding the difference between primiparous and multiparous women in their childbirth experience [37], only the former group was enrolled, which could be a research limitation. Women with a complicated pregnancy often report negative childbirth experience compared with women with uncomplicated pregnancy. Therefore, women with complicated pregnancy were excluded in this study [44]. Furthermore, CEQ has been developed based on the experience of low risk women with a healthy pregnancy and it assesses mothers’ perception of an uncomplicated childbirth experience [20]. This could be a limitation as the CEQ 2.0 is not suitable to use for high risk women. Considering that psychological problems [45, 46] or the use of antidepressants [47] could influence maternal-neonatal outcomes and may influence women’s perception of their childbirth experience, these groups also were excluded from the study. Including women with emergency caesarean section could have showed larger differences between known groups.

To measure the satisfaction level of childbirth experience in Iranian women, a reliable and valid tool is required. Satisfaction is known as a quality index and caregivers and policymakers can evaluate the quality of their services using this index, the current study can be used by them to investigate the quality of childbirth experience in clinical and research settings and enhance maternal satisfaction.

## Conclusion

The findings from this study suggest the translated CEQ, 2.0 is a reliable and valid measure of maternal childbirth experience and it can be used in clinical trials. However, additional research is warranted to design strategies tailored to the individual women’s needs and assess the effectiveness of the interventions to enhance women’s childbirth experience.

## Supplementary information

**Additional file 1.** English version of the CEQ 2.0.

**Additional file 2.** Farsi version of the CEQ 2.0.

## Data Availability

The datasets used and analysed during the current study are available from the corresponding author on reasonable request.

## Abbreviations

CEQ-2: Childbirth Experiences Questionnaire version 2.0
CFI: Comparative Fit Index
CTI: Childbirth Trauma Index
CVI: Content Validity Index
CVR: Content Validity Ratio
ICC: Intra Correlation Coefficient
KMO: Kaiser-Meyer-Olkin
LAS: Labour Agentry Scale
PAF: Principal Axis Factoring
RMSEA: Root Mean Square Error of Approximation
SRMSEA: Standardized Root Mean Square Error of Approximation
TLI: Tucker- Lewis Index
W-DEQ: Wijma Delivery Expectancy/Experience Questionnaire
