# Supplementary material for: Validation of the Iranian version of the childbirth experience questionnaire 2.0
Source: BMC Pregnancy Childbirth. 2019 Dec 4;19:465. doi: 10.1186/s12884-019-2606-y (PMC6894263; doi:10.1186/s12884-019-2606-y)
Supplement: Supplementary file 2 — Additional file 2. Farsi version of the CEQ 2.0. [file 12884_2019_2606_MOESM2_ESM.docx]

**مادر عزیز**

یکی از اهداف مراقبت زایمانی، اطمینان از کسب تجربه خوب از زایمان برای مادر می­باشد. هدف این پرسشنامه این است که بدانیم شما زایمان­تان را چگونه تجربه نمودید. پاسخ­های شما به همراه پاسخ سایر مادران به ما در ارزیابی مراقبت در طول زایمان کمک خواهد کرد.

دو راه برای گزارش تجربه­تان وجود دارد:

یا در داخل مربع­ها یا روی خط کشیده شده علامت بزنید.

مثال1:

در سوال زیر پاسخی را علامت بزنید که به دیدگاه شما نزدیک­ترین است.

سوال: من هر روز میوه می­خورم.

پاسخ­ها: کاملاً موافقم🞏 تا حدودی موافقم⌧ تا حدودی مخالفم🞏 کاملاً مخالفم🞏

مثال 2:

سوال: چقدر شما سیب دوست دارید؟

X

اصلاً میوه مورد علاقه ام

متشکریم از شما برای مشارکت و به اشتراک گذاشتن دیدگاه­هایتان

1. **مراحل زایمان همان‌طور که انتظار داشتم پیش رفت.**

کاملاً موافقم 🞏 تا حدودی موافقم 🞏 تا حدودی مخالفم 🞏 کاملاً مخالفم 🞏

1. **در طی مراحل زایمان، احساس می­کردم محکم و استوار هستم.**

کاملاً موافقم 🞏 تا حدودی موافقم 🞏 تا حدودی مخالفم 🞏 کاملاً مخالفم 🞏

1. **در طی مراحل زایمان، ­می­ترسیدم.**

کاملاً موافقم 🞏 تا حدودی موافقم 🞏 تا حدودی مخالفم 🞏 کاملاً مخالفم 🞏

1. **در طی مراحل زایمان، احساس می‌کردم از عهده­ی زایمان بر می‌آیم.**

کاملاً موافقم 🞏 تا حدودی موافقم 🞏 تا حدودی مخالفم 🞏 کاملاً مخالفم 🞏

1. **در طی مراحل زایمان، خسته (چه جسمی، چه روحی) بودم.**

کاملاً موافقم 🞏 تا حدودی موافقم 🞏 تا حدودی مخالفم 🞏 کاملاً مخالفم 🞏

1. **در طی مراحل زایمان، احساس رضایت داشتم.**

کاملاً موافقم 🞏 تا حدودی موافقم 🞏 تا حدودی مخالفم 🞏 کاملاً مخالفم 🞏

1. **احساس ‌کردم که خوب از عهده­ی مراحل زایمانی درآمدم.**

کاملاً موافقم 🞏 تا حدودی موافقم 🞏 تا حدودی مخالفم 🞏 کاملاً مخالفم 🞏

1. **ای کاش در طی مراحل زایمان، کادر پزشکی بیشتر به حرف‌هایم گوش می‌دادند.**

کاملاً موافقم 🞏 تا حدودی موافقم 🞏 تا حدودی مخالفم 🞏 کاملاً مخالفم 🞏

1. **هرچقدر که می‌خواستم می‌توانستم بلند شوم و قدم بزنم.**

کاملاً موافقم 🞏 تا حدودی موافقم 🞏 تا حدودی مخالفم 🞏 کاملاً مخالفم 🞏

1. **به اندازه­ای که می­خواستم در تصمیم‌گیری‌ها برای مراقبت و درمانم مشارکت نمودم.**

کاملاً موافقم 🞏 تا حدودی موافقم 🞏 تا حدودی مخالفم 🞏 کاملاً مخالفم 🞏

1. **با من و همسرم (یا همراهم) به گرمی و با احترام برخورد شد.**

کاملاً موافقم 🞏 تا حدودی موافقم 🞏 تا حدودی مخالفم 🞏 کاملاً مخالفم 🞏

1. **در طی مراحل زایمان، اطلاعاتی که نیاز داشتم را دریافت کردم.**

کاملاً موافقم 🞏 تا حدودی موافقم 🞏 تا حدودی مخالفم 🞏 کاملاً مخالفم 🞏

1. **ترجیح می‌دادم ماما در حین زایمان بیشتر بر بالین من حضور می‌داشت.**

کاملاً موافقم 🞏 تا حدودی موافقم 🞏 تا حدودی مخالفم 🞏 کاملاً مخالفم 🞏

1. **ترجیح می‌دادم ماما در طی مراحل زایمان دلگرمی بیشتری به من می‌داد.**

کاملاً موافقم 🞏 تا حدودی موافقم 🞏 تا حدودی مخالفم 🞏 کاملاً مخالفم 🞏

1. **ماما فضای آرامش‌بخشی به‌وجود ‌آورد.**

کاملاً موافقم 🞏 تا حدودی موافقم 🞏 تا حدودی مخالفم 🞏 کاملاً مخالفم 🞏

1. **ماما به من کمک کرد تا قدرت درونی­ام (اعتماد به نفسم) را دریابم.**

کاملاً موافقم 🞏 تا حدودی موافقم 🞏 تا حدودی مخالفم 🞏 کاملاً مخالفم 🞏

1. **برداشت من از مهارتهای طبی کادر پزشکی باعث شد که احساس امنیت خاطر کنم.**

کاملاً موافقم 🞏 تا حدودی موافقم 🞏 تا حدودی مخالفم 🞏 کاملاً مخالفم 🞏

1. **خاطرات مثبت زیادی از زایمان دارم.**

کاملاً موافقم 🞏 تا حدودی موافقم 🞏 تا حدودی مخالفم 🞏 کاملاً مخالفم 🞏

1. **خاطرات منفی زیادی از زایمان دارم.**

کاملاً موافقم 🞏 تا حدودی موافقم 🞏 تا حدودی مخالفم 🞏 کاملاً مخالفم 🞏

1. **یادآوری برخی خاطرات زایمانم به من احساس افسردگی می­دهد.**

کاملاً موافقم 🞏 تا حدودی موافقم 🞏 تا حدودی مخالفم 🞏 کاملاً مخالفم 🞏

1. **در کل، به نظرتان زایمان چقدر دردناک/ رنج­آور بود؟**


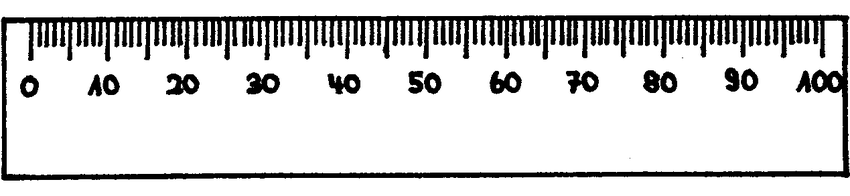


بدترین درد قابل تصور بدون درد

1. **کلاً ، در طول زایمان احساس می‌کردید که چقدر بر اوضاع کنترل داشتید؟**


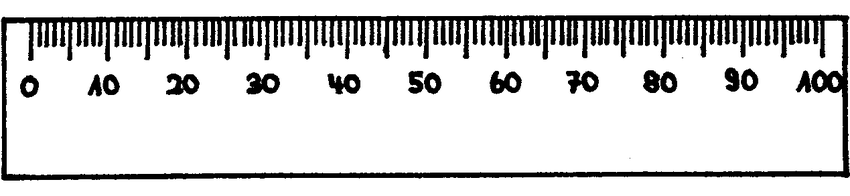


کنترل کامل بدون کنترل

1. **کلاً، در طول زایمان چقدر احساس آسایش خاطر می‌کردید؟**


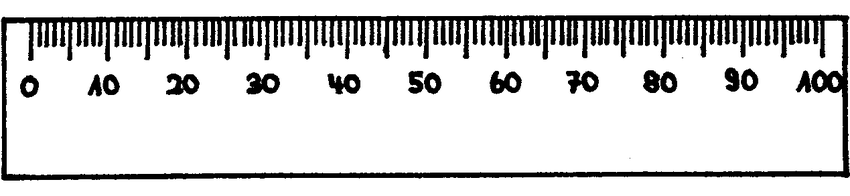


احساس آسایش­خاطر کامل عدم احساس آسایش خاطر

**پیشنهادات خود را در این کادر بنویسید.**

از همکاری شما سپاسگزاریم.
